# Diamonds in the Rough: Harnessing Tumor-Associated Myeloid Cells for Cancer Therapy

**DOI:** 10.3389/fimmu.2018.02250

**Published:** 2018-10-08

**Authors:** Emile J. Clappaert, Aleksandar Murgaski, Helena Van Damme, Mate Kiss, Damya Laoui

**Affiliations:** ^1^Myeloid Cell Immunology Lab, VIB Center for Inflammation Research, Brussels, Belgium; ^2^Lab of Cellular and Molecular Immunology, Vrije Universiteit Brussel, Brussels, Belgium

**Keywords:** tumor-associated dendritic cells, tumor-associated macrophages, myeloid-derived suppressor cells, tumor-associated neutrophils, cancer immunotherapy, tumor microenvironment

## Abstract

Therapeutic approaches that engage immune cells to treat cancer are becoming increasingly utilized in the clinics and demonstrated durable clinical benefit in several solid tumor types. Most of the current immunotherapies focus on manipulating T cells, however, the tumor microenvironment (TME) is abundantly infiltrated by a heterogeneous population of tumor-associated myeloid cells, including tumor-associated macrophages (TAMs), tumor-associated dendritic cells (TADCs), tumor-associated neutrophils (TANs), and myeloid-derived suppressor cells (MDSCs). Educated by signals perceived in the TME, these cells often acquire tumor-promoting properties ultimately favoring disease progression. Upon appropriate stimuli, myeloid cells can exhibit cytoxic, phagocytic, and antigen-presenting activities thereby bolstering antitumor immune responses. Thus, depletion, reprogramming or reactivation of myeloid cells to either directly eradicate malignant cells or promote antitumor T-cell responses is an emerging field of interest. In this review, we briefly discuss the tumor-promoting and tumor-suppressive roles of myeloid cells in the TME, and describe potential therapeutic strategies in preclinical and clinical development that aim to target them to further expand the range of current treatment options.

## Introduction

For a long time, tumors were thought to consist mainly of malignant cells, however this view changed in the past decades and tumors are now considered to behave as organ-like structures that contain besides cancer cells a large array of stromal cells. These tumor-infiltrating stromal cells comprise among others, immune cells, fibroblasts, pericytes, and endothelial cells, which closely interact with the cancer cells, forming the tumor microenvironment (TME) (1).

The interactions between the cancer cells and the immune system are initially hostile, resulting in many occasions in a successful eradication of the malignant cells (2). However, due to their rapid evolution, cancer cells can develop immune evasion mechanisms enabling them to avoid immune destruction (1). Furthermore, chronic inflammation caused by the tumor associated immune cells, secreting growth factors, cytokines, chemokines and reactive oxygen species, ultimately leads to an increased survival, growth and heightened rate of mutations in the DNA of the cancer cells (3). The presence of these tumor-promoting immune cells is often associated with an increased resistance to cancer therapies (4–8). Nevertheless, some of these tumor-associated immune cells still retain their anti-tumoral properties, the latter being suppressed by several factors produced in the TME (6, 9–12).

Deploying the immune system in anti-cancer therapies enables the specific targeting of (metastatic) cancer cell in the body expressing the specific tumor-associated antigens (TAAs). Most current immunotherapeutic approaches focus on lymphoid cells, particularly on the reactivation of pre-existing anti-tumoral T cells or adoptive transfer of tumor-specific T cells. In this respect, several immunotherapeutic strategies already made it to the clinic, such as CAR T-cell therapy or immune checkpoint inhibitors against PD-1, PD-L1, or CTLA-4, which are capable of re-invigorating T-cell responses in the TME (13–16). However, despite their success, *de novo* or acquired resistance against these therapies is widespread among patients (17), urging for the development of new immune therapies.

Targeting of tumor-associated myeloid cells, which abundantly infiltrate most solid tumors, might provide novel therapeutic approaches for cancer patients and is an emerging field of interest.

In this review, we briefly describe the role of several distinct tumor-associated myeloid cell subsets, i.e., macrophages, dendritic cells, neutrophils and MDSCs, with emphasis on their tumor-promoting and/or tumor-suppressive roles. Subsequently, the potential of myeloid cells in future cancer immunotherapy will be addressed.

## Macrophages

Referred to as “big eaters,” macrophages are one of the largest types of leukocytes, specialized in the phagocytosis of dead cells and pathogens. Besides their role in immune surveillance, macrophages are key players in tissue homeostasis maintenance and tissue repair (18). Macrophages are present in all tissues and originate from yolk sac macrophages, fetal liver monocytes and circulating monocytes that colonize the tissues in sequential waves (19, 20).

In tumors, macrophages can comprise up to 50% of the total hematopoietic compartment, negatively correlate with tumor progression and/or clinical outcome in many cancer types (21), with the majority of TAMs originating from circulating monocytes (22). However, certain studies, using orthotopic tumor models, showed that a fraction of the TAMs arises from the tissue-resident macrophages surrounding the tumor (23, 24). Recent evidence in several murine brain tumor models pointed out that the tissue-resident TAMs (microglia in this case) retained some of their tissue-specific traits, resulting in differential transcriptional profiles and activation states between microglia and monocyte-derived macrophages in the TME (23).

**Figure 1:**
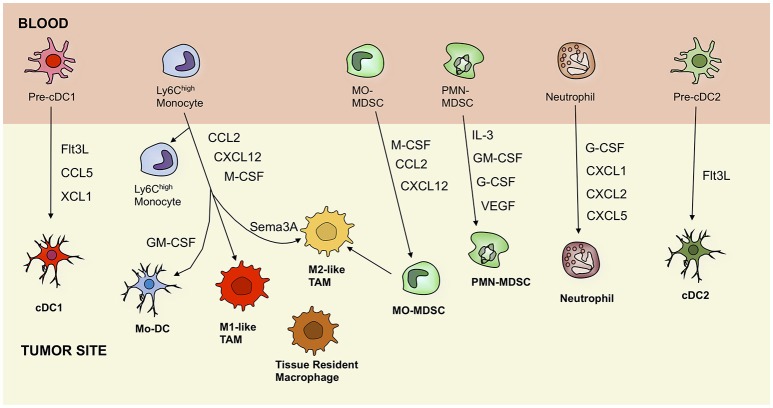
Ontogeny of tumor-associated myeloid cells, including dendritic cells, macrophages, monocytes, myeloid-derived suppressor cells, and neutrophils. Black arrows indicate recruitment pathways that are driven by secreted factors. cDC, conventional dendritic cell; Mo-DC, monocyte-derived dendritic cell; TAM, tumor-associated macrophage; MO-MDSC, monocytic myeloid-derived suppressor cell; PMN-MDSC, polymorphonuclear myeloid-derived suppressor cell; Flt3L, Fms-related tyrosine kinase 3 ligand; CCL5, C-C motif chemokine ligand 5; XCL1, lymphotactin; GM-CSF, granulocyte-macrophage colony-stimulating factor; CXCL12, C-X-C motif chemokine 12; M-CSF, macrophage colony-stimulating factor; Sema3A, semaphorin 3A; IL-3, interleukin 3; GM-CSF, granulocyte-macrophage colony-stimulating factor; G-CSF, granulocyte colony-stimulating factor; VEGF, vascular endothelial growth factor.

Importantly, multiple studies in mice showed that the TME was infiltrated with a heterogeneous monocyte-derived compartment and encompassed at least two molecular and functionally distinct TAM subsets, which populate different tumor microenvironments, namely a M1-like TAM subset, characterized by a more pronounced pro-inflammatory profile and higher expression of MHC-II and co-stimulatory molecules and a pro-angiogenic and immunosuppresive M2-like TAM subset (Figure 1) (10, 25, 26). The characteristics and emergence of these subsets are discussed elsewhere (7, 22, 27, 28).

It is, however, important to note that this M1/M2 dichotomy is an oversimplified representation of the vast range of activation states macrophages can adopt *in vivo* (29). Furthermore, recent studies in human tumors question the existence of distinct M1- and M2-like TAM subsets (30–32), indicating the need for a revised TAM nomenclature, which could be based on activation states, such as functional or metabolic programming, or by respecting a graded scale rather than separate entities, in line with the spectrum model of macrophage activity.

Two main TAM-based therapeutic strategies have recently gained interest in the fight against cancer: (i) depletion of TAMs through elimination of resident macrophages or inhibition of monocyte/macrophage recruitment to the TME and (ii) repolarization of immunosuppressive M2-like TAMs into anti-tumor M1-like TAMs. The first strategy is not the major focus of this review and is therefore only discussed briefly.

### Depleting TAMs through elimination of resident macrophages and/or inhibition of monocyte/macrophage recruitment

Several molecules were shown to efficiently deplete TAMs from the TME. The tunicate-derived chemotherapeutic molecule trabectedin demonstrates a cytotoxic activity against circulating monocytes and TAMs by activating the apoptotic pathway via TRAIL, which was successfully tested in several murine tumors models. This ultimately resulted in a decreased number of mononuclear phagocytes and an increased infiltration of anti-tumoral effector T cells in the TME (33, 34). Another group of drugs selectively targeting myeloid cells are bisphosphonates, such as clodronate-liposomes (35, 36) which induce the apoptotic pathway in TAMs as well. After liposome uptake, clodronate is released intracellularly and converted to a non-hydrolizable ATP analog, ultimately leading to the formation of pore openings in the mitochondrial inner membrane, eventually resulting in apoptosis. Finally, the conventional chemotherapeutic drug doxorubicin, which inhibits topoisomerase II, has been shown to significantly deplete TAMs in mice with orthotopic MMTV-Wnt1 triple-negative breast carcinoma, when encapsulated in nanoparticles specifically targeting TAMs, i.e., DOX-AS-M-PLGA-nanoparticles (37).

In the aforementioned treatment strategies, all TAMs are targeted, hence also depleting M1-like TAMs with potential anti-tumoral characteristics. Therefore, selectively depleting M2-like macrophages has gained interest. The identification of MMR as a marker for M2-like TAMs, residing in hypoxic tumor areas (10, 25), enables the visualization of these pro-tumoral macrophages for diagnostic purposes using anti-MMR Nanobodies *in vivo* (38, 39) and could potentially be coupled to toxic moieties for selective depletion of M2-like TAMs (40).

In order to prevent monocytes from maturing to tumor-promoting TAMs, the inhibition of monocyte recruitment to the TME can also be envisaged. One approach is to interfere with the CCL2/CCR2 axis, using an anti-CCL2 antibody (41) or bindarit, which inhibits CCL2 synthesis (42). Another important regulator of monocyte recruitment toward the TME is the CSF-1 receptor, whose inhibition leads to macrophage depletion in several murine and human tumors (43–45). Moreover, CSF-1R blockade using monoclonal antibodies or small molecule inhibitors not only leads to a reduced attraction of monocytes to the tumor, but also to the preferential differentiation of monocytes toward M1 TAMs, resulting in a higher intratumoral M1/M2 ratio in mice (46, 47). In addition, inhibition of either CCR2 or CSF-1R has been shown to decrease the chemotherapy-resistance of pancreatic tumors and to increase the T-cell mediated anti-tumor immune response in mice (48).

### Reprogramming of the TAM phenotype

**Figure 2:**
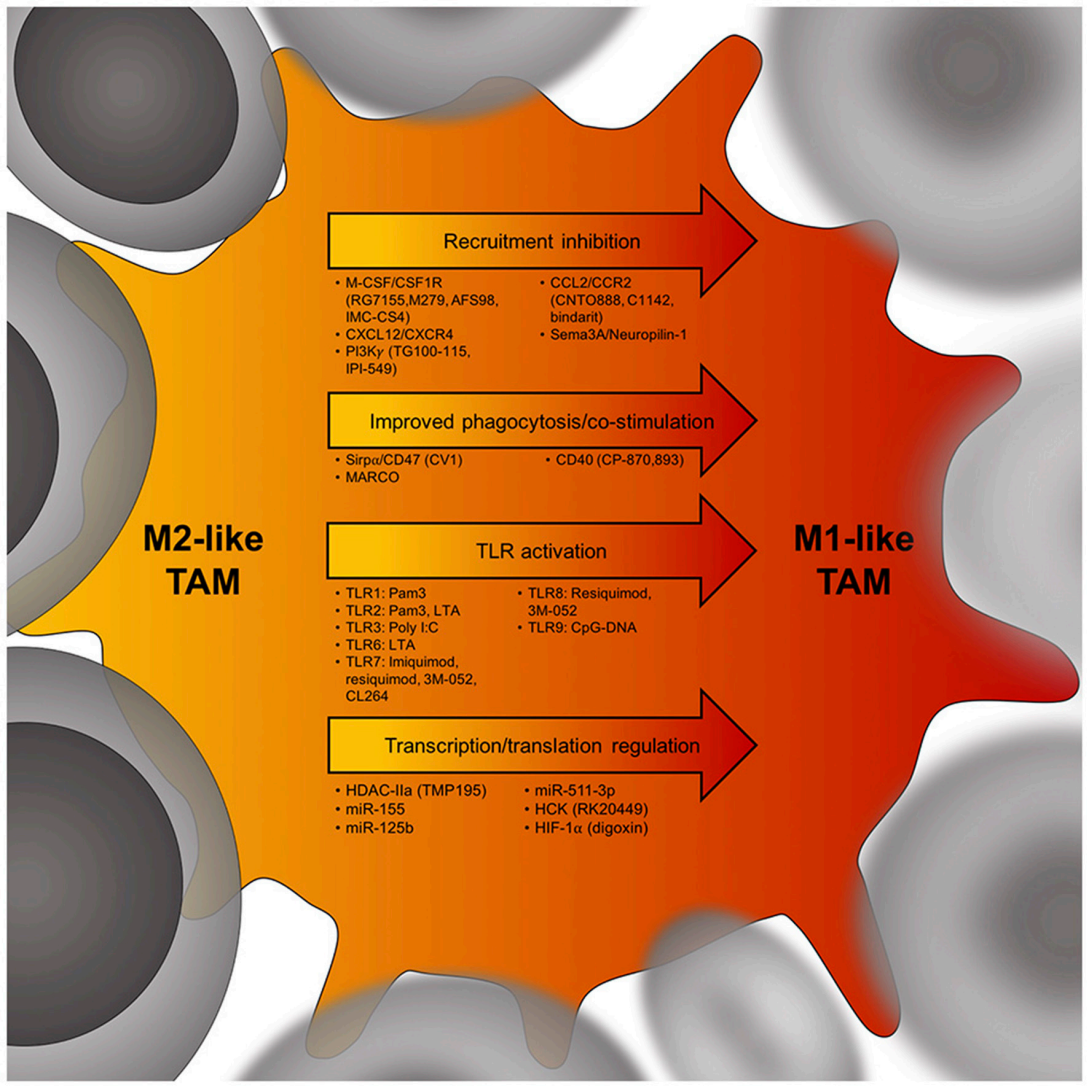
Potential targets to skew the TAM phenotype from an immunosuppressive M2-like TAM (yellow) to an anti-tumor M1-like TAM (red). Cancer cells are in gray, arrows indicate potential targets to induce a TAM phenotype shift within tumors. Below each arrow are specific targets that could influence M2-like TAM phenotypes. M-CSF, macrophage colony-stimulating factor; CSF1R, colony stimulating factor 1 receptor; CXCL12, C-X-C chemokine ligand 12; CXCR4, C-X-C chemokine receptor 4; PI3Kγ, phosphatidylinositol-3-kinase γ; CCL2, C-C chemokine ligand 2; CCR2, C-C chemokine receptor 2; Sema3A, semaphorin 3A; Sirpα, signal regulatory protein alpha; MARCO, Macrophage receptor MARCO; CD40, cluster of differentiation 40; TLR, toll-like receptor; HDAC-IIa, histone deacetylase IIa; miR155, microRNA 155; HCK, proto-oncogene HCK; HIF-1α, hypoxia-inducible factor 1-alpha.

Enforcing M1 programming of TAMs may reduce their tumor-promoting functions and help stimulate anti-tumor immunity, opening a new field in immunotherapy aiming at the repolarization of the M2-like TAMs to M1-like TAMs (Figure 2) (22, 49).

#### Inhibition of intracellular signaling pathways

A promising candidate for the repolarization of TAMs is the selective inactivation of phosphatidylinositol-3-kinase γ (PI3Kγ). This intracellular kinase has been shown to induce a transcriptional program via Akt and mTOR signaling ultimately leading to immune suppression in the TME (50). Inhibiting PI3Kγ genetically or via small molecules (TG100–115 or IPI-549) resulted in decreased tumor growth and prolonged survival in several murine tumor models, including head and neck squamous cell carcinoma, lung carcinoma and spontaneous breast carcinoma models. TAMs from mice lacking PI3Kγ showed increased levels of MHC-II and pro-inflammatory cytokines and were less immunosuppressive, which resulted in a restored CD8^+^ T-cell activation and cytotoxicity (50). In 4T1 breast carcinoma and B16-GM-CSF melanoma models, the inhibition of PI3Kγ by the small molecule inhibitor IPI-549, significantly improved the T-cell function and reduced immune suppression by increasing the M1/M2 ratio. Furthermore, in combination with PD-1 and CTLA-4, IPI-549 resulted in complete remission in 30% of the 4T1-bearing and 80% of B16-GM-CSF-bearing mice (51). Another key regulator of human M2 TAM gene expression is hematopoietic cell kinase (HCK), a member of the Src family kinases. Poh et al. showed that high HCK expression and activation correlated with a reduced survival of colorectal cancer patients and the preferential accumulation of M2-like TAMs respectively. Pharmacological inhibition or genetic reduction of HCK activity suppressed M2-like TAM activation and the growth of colon cancer xenografts, making HCK a promising target for cancer therapy (52). Finally, the inhibition of a group of histone deacetylases, HDAC class IIa, by a specific inhibitor, TMP195, reduced tumor burden and pulmonary metastasis by modulating the TAM phenotype in the murine MMTV-PyMT breast cancer model, and enhanced chemo-and T-cell checkpoint blockade therapy (53).

#### Toll-like receptor agonists

Toll-like receptor (TLR) agonists have been shown to be capable of stimulating the repolarization of M2-like TAMs toward M1-like TAMs, and therefore entail a promising future therapy. An example of such a ligand is the TLR7/8 agonist, 3M-052, which stimulated M2 to M1 polarization upon intratumoral injection. This approach resulted in a significant decrease of murine B16-F10 melanoma tumor growth through an elevated M1 phenotype-shifted macrophage infiltration with additional activation of CD8^+^ T cells, B cells, and pDCs. When used in combination with anti-PD-L1 and anti-CTLA-4 antibodies, cytotoxicity of TAMs and CD8^+^ T cells in the same melanoma model was potentiated (54). One of the TLR7 ligands, imiquimod, has been approved by the US Food and Drug Administration to topically treat early skin cancers. The use of imiquimod not only resulted in an inhibition of tumor growth, but also in complete regression of murine TSA mammary tumors, when used in combination with radiotherapy or low dose of cyclophosphamide (55). Another agonist of TLR7 and TLR8, namely R848 or resiquimod, loaded into β-cyclodextrin nanoparticles induced a functional re-orientation of the TME, in which the M2-like TAMs shifted toward a M1-like TAM phenotype, reducing tumor growth in multiple murine tumor models (56).

The use of a dsRNA analog, poly I:C, which is a potent TLR3 agonist, resulted in lewis lung carcinoma (LLC) regression in mice through the increased presence of tumor-suppressive M1-like TAMs (57). Strikingly, already 1 h after intraperitoneal injection, TNF-α levels increased, leading to the subsequent decrease of LLC tumor growth (57). The TLR9 agonist CpG-DNA, was able to induce reprogramming of TAM from a M2-like to a M1-like phenotype, alone or in combination with an anti-IL-10R Ab when injected intratumorally in 4T1 breast tumor-bearing mice (58). In addition to the repolarization of TAMs, this molecule was able to stimulate a cytotoxic T-cell response in the murine EG7-OVA lymphoma model (59).

Aside from the aforementioned strategies, combination therapies using both TLR agonists and immune checkpoint inhibitors have also been shown to be beneficial. Intratumoral injections of TLR7 and TLR9 agonists [1V270 and SD-101(CpG), respectively] alongside with systemic administration of anti-PD-1 mAbs successfully suppressed tumor growth in murine models of head and neck squamous cell carcinoma (60). Regression was not only observed at the primary tumor site, but distant tumors were suppressed as well, with a clear increased ratio of M1-like to M2-like TAMs (60). In addition, the efficacy of anti-PD-1 treatment in athymic nude mice implanted with human osteosarcoma relied on the presence of macrophages in the tumor. As such, anti-PD-1 treatment led to a higher activation of M1 macrophages due to repolarization from M2 TAMs, likely due to STAT3 signaling blockade (36).

Müller et al. tested a whole panel of TLR agonists with or without co-administration of IFNγ in an *in vitro* cancer cell growth inhibition assay using bone marrow-derived macrophages. Their results pointed out that IFNγ and the TLR agonists [LPS, poly(I:C), TLR1/2 agonist Pam3, TLR2/6 agonist LTA, TLR7 agonist CL264, and TLR9 agonist CpG] acted in synergy to induce macrophage tumoricidal activity and production of both NO and pro-inflammatory cytokines. These results suggest that IFNy secretion in the TME may be an important factor that determines the effectiveness of TLR agonists (61).

Analogous to the activation of TLRs, bacterial species can be inoculated in the TME, resulting in acute inflammation and M1-like TAM activation. Bacteria mediated tumor therapy has been extensively reviewed elsewhere (62, 63).

#### TAM repolarization and miRNAs

One of the post-transcriptional regulators that mediate differentiation of monocytes into either M1-like or M2-like TAMs are miRNAs, small non-coding pieces of RNA of approximately 20–25 nucleotides. While their exact functions in macrophage polarization are yet to be fully elucidated, some have already gained interest for future therapies.

A gain of function study showed that overexpressing miR-155 in M2-activated macrophages led to repolarization of these cells into proinflammatory M1-like macrophages (64). Through the regulation of FGF2 expression, miR-155 was able to decrease tumor progression, making it a potential target in future immunotherapy (65). Overexpression of another miRNA, namely miR125b, using a viral vector, proved to promote the M1-like activation, leading to an increased cytotoxic activity against EL4 cancer cells *in vitro* and *in vivo* (66). Transfecting miR125b using CD44 targeting nanoparticles led to a 6 fold increase in the M1/M2 ratio in a mouse model of non-small cell lung cancer (67). Another strategy involved the enforced expression of miR-511-3p, which is encoded by MRC1 genes, in TAMs, resulting in a decreased protumoral gene signature of MCR1 (MMR)^+^ TAMs and inhibited murine LLC tumor growth (68).

Finally, the importance of miRNAs in the differentiation of macrophages in the TME was demonstrated by Baer et al. in mice, where the inactivation of the miRNA-processing enzyme DICER in TAMs promoted the intratumoral expansion of M1-like TAMs, with a pronounced IFN-γ/STAT1 transcriptional signature and the concurrent demise of M2-like TAMs. The TAM's phenotype switch was associated with enhanced tumor infiltration by cytotoxic T-cells (CTLs) and IFN-γ production, MC38 tumor inhibition and, importantly, increased tumor responsiveness to PD1 checkpoint blockade (69).

#### Tumor vascularization and TAM repolarization

The high consumption of nutrients and oxygen by the cancer cell mass demands a constant and sufficient intratumoral blood flow. To that end, angiogenesis is promoted in the TME through excessive secretion of pro-angiogenic factors, such as vascular endothelial growth factors (VEGFs). However, this uncontrolled tumor vascularization leads to imperfect and leaky blood vessels, promoting metastatic dissemination and intratumoral hypoxia (70). For a long time, the preferred strategy was to further disrupt the vessel composition in order to starve cancer cells. However, this resulted in a more aggressive tumor and often increased metastatic outgrowth. These findings suggest that the opposite strategy, i.e., improving the functionality of the tumor vasculature (also termed vessel normalization), might be more beneficial to the patient (71). Both aforementioned strategies also have their impact on the TAM composition in the TME.

Although intratumoral vessel disruption strategies lead to more aggressive cancer progression and metastasis, their use has also been shown to elicit macrophage phenotype skewing, demonstrating potential tumor-suppressive functions. An example of this strategy is the vascular disrupting agent 5,6-di-methylxanthenone-4-acetic acid, DMXAA, which was shown to induce the repolarization of M2-like TAMs to an M1-like phenotype in a mouse model of non-small cell lung cancer (72). However, vascular disruption also resulted in increased hypoxia, leading to the subsequent activation of HIF-1α, resulting in a more aggressive cancer phenotype. Accordingly, inhibition of HIF-1α using digoxin was synergistic with DMXAA and led to stronger inhibition of tumor growth and metastasis of murine B16-F10 melanoma than DMXAA or digoxin alone (73). However, the direct effect of the treatment on M1-like TAMs remains to be elucidated. Another vascular disruption agent which showed such characteristics, is Z-GP-DAVLBH, which induced the secretion of GM-CSF and the skewing of M2-like to M1-like TAMs in hepatocellular carcinoma and breast cancer xenografts, leading to higher rates of cancer cell apoptosis (74).

Vessel normalization strategies, such as the inhibition of ANG2 and VEGF, also have the potential to induce repolarization of TAMs. In murine and human glioblastoma models, a bispecific antibody against ANG2/VEGF was shown to induce prolonged survival through reprogramming of TAMs from a M2 to a M1 phenotype (75). Similar observations were made by other research groups when using peptibodies inhibiting both the ANG2 and VEGF receptors or a bispecific antibody inhibiting ANG2 and VEGF themselves (76–78). Finally, another factor capable of promoting TAM repolarization and vessel normalization is histidine-rich glycoprotein (HRG), which is generally only expressed in low levels in the TME. A gain-of-function experiment, transducing *HRG* in T241 fibrosarcoma, Panc02 pancreatic carcinoma and 4T1 breast carcinoma models, showed reduced growth mediated by an increased presence of M1-like TAMs (79).

#### Alternative strategies increasing M1/M2 ratios

The use of antibodies in the reprogramming of TAM ratios has also proven successful when agonistic anti-CD40 antibodies were administered in combination with gemcitabine, resulting in tumor regression in both mice and human patients with pancreatic ductal carcinoma (80). In this study, tumor regression did not seem to depend on gemcitabine or T cells, but on the presence of activated macrophages (80). Interestingly, CD40 agonist antibodies have been shown to induce tumoricidal properties in macrophages and to promote the maturation of antigen presenting cells, making them an ideal choice for combination therapies with immune checkpoint inhibitors (81, 82).

Similarly, antibody-mediated targeting of other surface receptors such as the pattern recognition receptor MARCO on TAMs resulted in altered macrophage polarization and a reduction in tumor growth and metastasis in a mouse model of breast cancer (83).

Moreover, the intratumoral localization of TAMs within the TME can also be targeted, as hypoxia or increased lactate levels, induces a proangiogenic, immunosuppressive TAM phenotype (25, 84). Therefore, retaining the TAMs in normoxic regions in order to prevent M2-like TAM differentiation could prove to be a valuable strategy. Blunting the Sema3A/Neuropilin-1 pathway through genetic deletion of neuropilin-1 in mice demonstrated decreased migration of TAMs to the hypoxic regions, resulting in a strengthened immune response (85).

A strategy which does not involve direct reprogramming of the macrophages, comprises the blockade of the “don't eat me” signal CD47, which is overexpressed by most cancer cells, or its corresponding receptor on macrophages, signal regulatory protein α (SIRPα). SIRPα interacts with CD47, leading to the downregulation of phagocytotic programs. Hence, inhibition of CD47 signaling increases phagocytosis by TAMs (86). These observations prompted clinical trials with anti-CD47 antibodies, which are currently ongoing (87). Alternatively, the administration of a CD47 antagonist, namely the engineered SIRPα variant CV1, in combination with other molecules inducing phagocytosis, such as IgG4, significantly increased the phagocytic activity of macrophages and suppressed tumor growth of xenografts in mice (88).

In the search for molecules that could prolong survival of cancer patients, the anti-malaria drug chloroquine was tested. As a small molecule with a long clinical record which is affordable for clinical use, it was proven to induce repolarization of M2 macrophages toward the tumoricidal phenotype in the murine B16 melanoma model, showing promising results for future clinical trials (89). Another experimental treatment involved the use of a copper chelate to trigger activation of mitogen-activated protein (MAP) kinases via ROS generation. This led to the upregulation of IL-12 and IFNγ production and subsequent repolarization of the tumor-promoting M2 TAMs in the Ehrlich ascites carcinoma model (90).

Overall, repolarization of TAMs appears to be a viable approach based on a large number of preclinical studies using a wide range of therapeutic agents, however, the safety and clinical efficacy of most therapies still remain to be investigated.

## Dendritic cells

The bridge between the adaptive and the innate immune system is formed by antigen presenting cells (APC) such as dendritic cells (DCs). DCs are specialized in the processing of foreign antigens and their subsequent presentation, alongside relevant costimulatory molecules, to effector cells of the adaptive immune system in secondary lymphoid organs, such as the lymph nodes. Eventually, these effector cells, being cytotoxic CD8^+^ T cells, helper CD4^+^ T cells and B cells, will differentiate and engage in the elimination of those cells expressing the foreign antigen.

### DC identity

DCs can be subdivided into two distinct specialized lineages, being the conventional/myeloid DCs (cDCs) and the plasmacytoid DCs (pDCs) (Figure 1). Both in mice and in humans, the existence of two cDC populations was demonstrated: CD8α^+^ or CD103^+^ cDC1s and CD11b^+^ cDC2s in mice and CD141^+^ (or BDCA3^+^) cDC1s and CD1c^+^ (or BDCA1^+^) cDC2s in humans (91–93). Finally, a population of monocyte-derived DCs (Mo-DCs) is also distinguished both in mice and in humans, as part of the myeloid DC lineage (94, 95). Based on single-cell RNA sequencing data, six populations were distinguished in human peripheral blood during steady-state. Two populations were identified as two cDC2 CD1c^+^ subpopulations and one was appointed as a new unidentified population of AXL^+^SIGLEC6^+^ cells (95). The latter was shown to stimulate both CD8^+^ and CD4^+^ T-cell proliferation in a way similar to cDCs, while they express several pDC markers as well. Other populations resembled the CLEC9A^+^ cDC1, the CD1c^−^CD141^−^CD11c^+^ monocyte-derived DCs (mo-DCs) and pDCs (95).

The cDC1s were shown to interact mainly with CD8^+^ T cells to induce potent CTL responses, while cDC2s can induce Th2 or Th17 responses, through presentation of tumor associated antigens (TAAs) on their MHC-II complexes (12, 94, 96). Plasmacytoid DCs engage in the secretion of type-I IFN, IL-6, and TNF-α and in this way interact with cDCs, T cells and B cells in order to counteract infections (97). Mo-DCs arise from monocytes during inflammation, and could hence be seen as an activated type of macrophages, and have been shown to express immunosuppressive properties (94, 98).

Within the TME, DCs were originally described as immunosuppressive cells, characterized by an immature differentiation state, marked by a high antigen uptake and inadequate antigen presentation (99). These DCs are thought to enable further tumor growth and are therefore referred to as tolerogenic or regulatory DCs (9). The factors, responsible for the shift and maintenance of the immunosuppressive TADC phenotype are described in Conejo-Garcia et al. (100), while the mode of regulation by which these TADC exhibit immune suppression is reviewed in Keirsse et al. (9). Interestingly, the coexistence of distinct cDC subsets with anti-tumoral properties was recently shown in several murine models and patient biopsies (94, 101, 102). In this review, we focus on the anti-tumoral properties of TADCs and the strategies deploying TADCs for immune therapy.

### DC vaccination strategies

DCs display a high potential for the development of immunotherapy, considering their ability to induce a potent anti-tumoral immune response involving the activation of anti-tumoral T cells (CD8^+^ and CD4^+^). These anti-tumoral T cells are not only capable of fighting the primary tumor but also their metastatic lesions and potential recurrence. The development of DC-based immunotherapy led to the emergence of DC-based vaccines, whereby DCs are activated through: (i) *ex vivo* incubation with a maturation cocktail containing cytokines and/or TLR agonists, (ii) the administration of TAAs *ex vivo* or *in vivo*, or (iii) intra-tumoral administration of immuno-stimulatory molecules that activate TADCs. These DC-based vaccines can be categorized into distinct generations based on when they were first applied in the clinic (103), and are intensively studied in (pre-)clinical trials for their application in future cancer immunotherapy (104).

First generation DC-vaccines involved Mo-DCs that were isolated from the blood of the patient or that were generated *ex vivo* (105). However, these DCs were not matured any further using maturation cocktails, but were incubated *ex vivo* with synthetic TAAs or tumor lysates. The fact that these cells remained largely immature explains their inability to elicit a strong and durable anti-tumoral response (105). Therefore, during development of the second generation of DC vaccines, Mo-DCs were maturated using a maturation cocktail containing both cytokines and TAAs, successfully activating the APC properties of the dendritic cells (106). The first DC-based vaccination strategy that received FDA approval, being Sipuleucel-T in 2010, which specifically acts against metastatic castration-resistant prostate cancer (CRPC) is an example of a second-generation DC vaccines. In this strategy, immature dendritic cells were isolated from the blood and incubated with a fusion protein PA2024, which contains GM-CSF, a prostate antigen and prostate acid phosphatase (107).

The delivery of antigens to DCs can be performed *in vivo* or *ex vivo* through several strategies listed by Garg et al. (104). The genetic modification of dendritic cells for more efficient vaccine activity using mRNA and siRNA but also viral transfection and fusion with malignant cells has been reviewed in Abraham et al. The application of this approach is generally to improve cancer cell-targeting, however it also helps in reducing the effect of tumor-mediated immunosuppression on the reinjected DCs (108).

Recent developed strategies aim for the *in vivo* loading of TAAs, without the need for additional *in vitro* maturation or treatment. This involves the *in vivo* injection and targeting of TAAs to dendritic cells (109). However, recent research in mice demonstrated the potential of using TADCs (cDC1 and cDC2) isolated directly from the primary tumor (94). The reinjection of these TADCs, which took up the TAAs *in vivo*, led to the onset of immunological memory. Prophylactic vaccination with tumor-derived cDC1s elicited an anti-tumor CTL response in B16-OVA melanomas, whereas cDC2 vaccination reduced LLC-OVA tumor growth through a Th17 response (94). It remains to be elucidated, whether tumor-derived DCs can induce an efficient memory response against tumor antigens in cancer patients.

The antigen-loading can also be induced by immunogenic cell death (ICD), in which cancer cell apoptosis is induced, resulting in the release of antigens (110). As such, photodynamic therapy, which generates ROS-mediated ER stress, induced immunogenic apoptosis in cancer cells characterized by phenotypic maturation and functional stimulation of dendritic cells as well as induction of a protective antitumor immune response (111). This strategy has been shown to increase the survival of high grade glioma-bearing mice when activated DCs were administered as a prophylactic vaccine (110). In combination with conventional chemotherapy (temozolomide), the ICD-based DC vaccines enabled an increased survival and complete tumor rejection (110). Similarly, the treatment of cancer cells with high hydrostatic pressure enhanced the *in vitro* uptake and presentation of TAA. This DC-based vaccine inhibited tumor growth of TC1 tumors in mice when combined with docetaxel chemotherapy (112).

### Combining DC-vaccination with co-stimulatory molecules

Success rates of DC-based vaccination strategies can be improved through co-injections of stimulatory molecules, like TLR agonists or CD40 agonists, which can enhance the antigen presenting function of TADCs (109). *In vivo* TAA presentation by TADCs can be induced through the intratumoral injection of TriMix mRNA, containing mRNA coding for the CD70 costimulatory molecule, the activation stimulus CD40L, and constitutively active TLR4 (113). Administration of DCs electroporated with TriMix mRNA and a melanoma antigen (gp100, tyrosinase, MAGE-A3 or MAGE-C2 fused to DC.LAMP) demonstrated durable clinical benefit in clinical trials involving patients with advanced melanoma when combined with the CTLA-4 inhibitor ipilimumab (114, 115). CD40 signaling induces important changes in DCs, including the induction of antigen presentation and upregulation of MHC- II and co-stimulatory molecules CD80 and CD86 (116). The use of an agonistic anti-CD40 antibody proved to successfully activate cDC populations (117), making it an interesting adjuvant for DC vaccination. Moreover, CD40 and TLR agonists act synergistically and the combination of these immunostimulants can significantly suppress B16-F10 tumor growth in mice (118). Aside from CD40L, Fms-like tyrosine kinase 3 receptor ligand (Flt3L), a potent growth factor typically associated with DC development (119), was also suggested as an interesting candidate for the maturation of the TADCs. In this respect, co-administration of an adenoviral vector encoding Flt3L (pAd-Flt3L) and cell lysate of the colon cancer model CT26 into the footpad of the mouse prior to subcutaneous injection at the same location with CT26 resulted in the successful priming of both cDCs and pDCs, enabling tumor regression (120).

Other promising candidates are the TLR7/8 agonist FSME, which stimulates pDCs, and GM-CSF, which promotes myeloid-derived DC maturation. Administration of FSME or GM-CSF prior to DC vaccination in melanoma patients resulted in the induction of potent anti-tumor immune responses (121, 122). Also, intratumoral injection of GM-CSF secreting whole cell tumor cell vector (GVAX) formulated with the TLR4 agonist LPS showed potent induction of DC maturation and therapeutic efficacy in CDT26-tumor bearing mice (123).

Interestingly, Salmon et al. observed significant activation of CD103^+^ DC progenitors (cDC1s) in the TME of the B16-OVA breast cancer model in mice after systemic administration of Flt3L, alongside intratumoral injection of the TLR3 agonist poly I:C (124). This therapy also enhanced the response to anti-PD-L1 therapy and BRAF inhibition (124), opening up possibilities for combination therapy with both immune checkpoint inhibitors and DC vaccination. The TLR3 agonist poly I:C was also employed in the development of a nanovaccine which was loaded with poly I:C, together with small interfering RNA (siRNA) against STAT3 and the ovalbumin antigen. The use of this carrier induced a significant tumor regression of B16-OVA tumors in mice with an increase of TADCs and decrease of immunosuppressive cells in the tumor draining lymph nodes (125). Similarly, a poly(lactic-co-glycolic acid) nanoparticle loaded with poly I:C and coated with a CD40 agonist antibody was directed toward CD40 expressing CD11c^+^CD11b^+^F4/80^−^ DCs *in vivo*, resulting in prolonged survival of B16-OVA-tumor bearing mice (126). While the use of nanocarriers, which facilitate the *in vivo* delivery of antigens to dendritic cells, represents a promising strategy, it still requires validation through clinical trials in human patients.

The immune system in cancer patients is not only suppressed in the TME, but is altered systemically, whereby activation of immune cells in the draining lymph nodes is also counteracted (127). Intradermal injection of combined CpG-B/GM-CSF administration resulted in enhanced *in vivo* maturation and frequencies of cDCs in the lymph nodes of patients with stage I-II melanoma and these cDCs displayed increased cross-presentation capacities after *ex vivo* culture (128), suggesting the potential of CpG-B/GM-CSF as a possible new combination partner for DC-based immunotherapies against metastatic spread. Given the existence of systemic immune suppression, tumor-specific CD8^+^ T-cell responses mediated by DC-vaccinations can be maximized using a multi-site injection strategy. This approach has been applied using a replication-deficient adenovirus serotype 5-vectored cancer vaccine. This vaccine specifically targeted the dopachrome tautomerase antigen in melanoma and led to an increase in systemic TAA-specific T-cells. Hence, the use of multi-site injections could also show potential in future DC vaccination strategies (129). Since systemic activation of the immune system in cancer is considered as beneficial for the efficacy of immunotherapy (130), systemic activation of DCs leading to an anti-tumoral immune response is another field of investigation. With the administration of RNA-lipoplexes, lipid carriers containing RNA encoding antigens (ovalbumin, gp70), efficient systemic uptake by DCs led to maturation and induction of effector/memory T-cell responses resulting in IFNα-mediated tumor inhibition (131).

### Other DC-based strategies

The amount of cDCs that can be recovered from the circulation or tumors can be critical for enabling DC-based vaccination strategies. The accumulation of cDC1s appears to depend, besides Flt3L signaling, also on natural killer (NK) cells that secrete CCL5 and XCL1, which are potent cDC1 chemoattractants. Böttcher et al. proved in mice that the production of PGE_2_ by the tumor impaired NK cell chemokine secretion and cDC1 chemokine receptor expression, leading to a decreased recruitment and anti-tumoral action of cDC1s in the tumor (132). The discovery of the CCL5-XCL1 mediated attraction of cDC1s into the TME, opens possibilities for future cancer immunotherapy, employing injection of these chemokines intratumorally alongside intranodal injection of TAA-loaded cDC1s. Efficient cross-presentation of tumor antigens to CD8^+^ T cells by cDC1s is a major determinant of antitumor immune responses, thus therapeutic enhancement of this activity in the TME and the lymph nodes is of great interest (133).

A recent strategy shown to induce a cytotoxic T-cell response and NK cell activation, comprises the use of DC-derived exosomes, which contain functional MHC complexes (both MHC-I and-II) including costimulatory molecules (134) and demonstrated to successfully slow down tumor growth and increase a anti-tumoral immune cell infiltration when injected intravenously in a murine hepatocellular carcinoma model (135).

Lastly, low-dose administration of chemotherapeutic agents such as cyclophosphamide or paclitaxel was shown to enhance DC maturation, migration and function (136). Administration of immature DCs in the peritumoral environment of head and neck cancer patients together with low-dose cyclophosphamide and docetaxel as well as a multi-cytokine inducer OK-432, reduced immunosuppression and enhanced T-cell immunity, as a consequence of DC maturation (137). Combination therapy with low-dose cyclophosphamide and DC vaccination also demonstrated to reduce the tumor-induced immune suppression in patients with mesothelioma (138).

## Neutrophils

Neutrophils are highly phagocytic innate immune cells that make up 50–70% of all circulating leukocytes and live 5 to 8 h in the blood (139). In the steady-state, neutrophils are retained in the bone marrow through the secretion of CXCL12 by osteoblasts. Upon infection and tissue damage, endothelial cells secrete CXCL1 and CXCL2, the major chemokines involved in the recruitment of the neutrophils, which are both recognized by CXCR2 (140). Another important player, counteracting retention of the neutrophils in the bone marrow is G-CSF (141). This growth factor does not only play an important role in the activation of neutrophils, but is also a major actor in the infiltration of neutrophils into the TME (142). When neutrophils migrate to the site of threat, they become activated and recruit other types of immune cells, leading to acute inflammation. When encountering harmful microorganisms, neutrophils will engage in three ways: (1) phagocytosis, (2) degranulation, and (3) release of neutrophil extracellular traps (NETs) (3).

Being the largest group of circulating white blood cells in the body, neutrophils play a substantial role in the interaction with malignant cell growth. Neutrophils in the TME, also called tumor associated neutrophils (TANs), tend to live longer (up to 17 h) under the influence of different signals present in the tumor, such as G-CSF and hypoxia (143). In humans, neutrophils are identified through their expression of the cell surface markers CD66b, CD15, CD16, and CD10 (144). Additionally, the lectin-type oxidized low-density lipoprotein receptor-1 (LOX1) is a potent marker which can be used to separate them from polymorphonuclear-MDSCs (PMN-MDSCs) (145), which can be described as immature neutrophils and are LOX1^+^ (see section Myeloid-Derived Suppressor Cells). Besides these surface markers, it is also possible to identify TANs based on high expression of typical neutrophil-associated enzymes such as the serine protease neutrophil elastase (NE) (146) and myeloperoxidase (MPO) (147).

Peripheral blood neutrophil to lymphocyte ratio can be used in a clinical context as a prognostic biomarker and is associated with a poor overall survival in many solid tumors (148–150). TAN infiltration is mediated via the known neutrophil recruiting chemokines, being CXCL1, CXCL2, and CXCL5, secreted by cancer cells (Figure 1) (139, 151). Strikingly, it has also been shown that some malignancies can stimulate osteoblasts to upregulate the production and recruitment of tumor-promoting neutrophils (152). When neutrophils are initially recruited to the tumor, they appear to exhibit anti-tumoral properties and only over time become tumor-promoting, through the action of several factors secreted in the TME (147, 153). The initial tumor killing capacity of neutrophils is illustrated by an *in vitro* study, where Yan et al. demonstrated that neutrophils derived from the peripheral blood of healthy individuals were able to kill four different human cancer cell lines (154). Neutrophils, whose phenotype has switched toward tumor promotion facilitate metastasis (155), angiogenesis via secretion of proangiogenic factors, such as MMP9 and VEGF (156, 157) and immunosuppression either directly or through the recruitment of regulatory T cells (Tregs) (153).

### TAN repolarization

The tumor-suppressive properties of TANs appear to be reversible, based on mouse studies, leading to an anti-tumor neutrophil phenotype often termed N1 as opposed to the pro-tumor N2 phenotype, analogous to the M1/M2 concept used to describe the extremes of macrophage polarization. One of the central signals in the TME that induces the pro-tumor TAN phenotype appears to be TGFβ, which induces the expression of CXCL1, VEGF, and MMP9, which are all factors leading to a more persistent tumor growth (158). Accordingly, using a TGFβ receptor inhibitor SM16 led to a suppression of tumor growth by the anti-tumor N1-like TANs in mice, which expressed TNFα, MIP1α, H_2_O_2_, and NO, ultimately being cytotoxic to cancer cells (159). Other molecules, such as type I IFNs can also induce the shift toward an anti-tumor TAN phenotype (157, 160, 161). Therefore, it might be interesting to further explore the generation of N1-like TANs as a potential new immunotherapy approach.

### Increasing anti-tumoral TAN infiltration

The creation of an acute inflammatory response instead of the wound-healing and tissue-repair response characteristic for the TME (162), could also prove to be a promising strategy. The ample evidence pointing toward the potential of neutrophils to serve as anti-tumor effectors was reviewed by Souto et al. (163). One of the approaches to enhance anti-tumor neutrophil infiltration could be radiotherapy. Infiltration of neutrophils producing large amounts of reactive oxygen species following radiotherapy were reported to exhibit a potent anti-tumor effect by inducing oxidative damage and apoptosis in cancer cells in several mouse tumor models (142). Therapies aiming to induce systemic neutrophil expansion (e.g., G-CSF) in combination with agents that promote the generation of anti-tumor neutrophils (e.g., TGFβ targeting) might act synergistically, and induce greater cytotoxicity in the tumor. It remains to be investigated, whether such combination therapies could be beneficial considering the largely negative effect of G-CSF administration on disease outcome. Until now, G-CSF has been administered to induce neutrophil expansion in order to help patients recover from chemotherapy-induced neutropenia (141). However, many studies have shown negative effects of this growth factor on disease outcome (141, 164, 165) and suggest G-CSF neutralization as a target for immunotherapy (166, 167). Accordingly, although administration of G-CSF in mice expanded neutrophils, it failed to induce a cytotoxic neutrophil response (168). Furthermore, in mice, G-CSF has also been shown to inhibit neutrophil migration through inhibition of CXCR2 (169). Therefore, other signaling molecules, such as intratumoral delivery of IL-8 could be used to stimulate neutrophil infiltration in order to induce acute inflammation and consequential inhibition of tumor growth (170, 171). A wide range of chemokines haven been shown to induce neutrophil cytotoxicity *in vitro*, including CCL2, CCL3, CCL5, CXCL1, CXCL12, and CXCL16, therefore approaches that increase the secretion of these factors in the TME might also prove to be beneficial (168). Inhibition of certain receptor tyrosine kinases (cMET, VEGFR2, RET, KIT, AXl, and FLT3) using a promiscuous small molecule inhibitor, cabozantinib, has also led to higher neutrophil infiltration into the tumor. Ultimately, these neutrophils induced a highly effective eradication of murine prostate cancer (172). The precise mechanism behind the higher infiltration is not entirely clear, as (1) the exact RTK targeted is not yet identified (172) and (2) the application of cabozantinib inhibited tumor infiltration of immature neutrophils in another study on a more aggressive type of prostate cancer (173).

### Inhibiting immunosuppressive TAN infiltration

In contrast to inducing an acute form of inflammation via an increased neutrophil infiltration, in the last decade, many researchers have focused on developing strategies to inhibit neutrophil recruitment to the TME. This is due to the finding that neutrophils often acquire an immunosuppressive phenotype upon infiltration of the TME. One strategy in preclinical studies was the inhibition of the general neutrophil recruitment pathway, involving the blockade of the IL-8/CXCR1/CXCR2 axis (140) with CXCR2 antagonists (174) or anti-IL8 antibodies (156). Moreover, there are indications in mice that the inhibition of RTK MET can also result in decreased tumor infiltration of immunosuppressive neutrophils in response to adoptive T-cell therapy leading to enhanced anti-tumoral T-cell function (175). However, in certain murine tumor types, inhibition of MET has been reported to diminish infiltration of antitumor neutrophils, resulting in increased tumor growth and metastasis (176).

Another possible strategy could be the induction of reverse migration or retrotaxis of TANs out of the TME in the bloodstream, lowering the abundance of TANs in the tumor microenvironment. These reverse migrated TANs could then possibly induce a more systemic anti-tumor response by antigen presentation or direct T-cell stimulation (177, 178). Therapeutic induction of neutrophil reverse migration has only been witnessed in case of wound-induced inflammation, however the development of reverse migration-inducing drugs might potentially open up opportunities for future cancer therapies (179). Two signaling pathways involved in reverse migration have already been discovered, namely the redox-regulated Src family kinase signaling (180) and the leukotriene B4-neutrophil elastase axis (181).

### Other TAN-based strategies

Other strategies that have been investigated to target neutrophils in the TME involve inhibition of enzymes and mediators known to induce pro-tumorigenic properties, namely NE (182), a2 isoform V-ATPase (146), arachidonate 5-lipoxygenase (155), IL-23 (139), and IL-17 (183). Again, the latter can also promote anti-tumor activities (158), illustrating that the role of TANs appears to be highly context-dependent, determined by the histological origin and stage of the tumor as well as the therapies applied in the treatment.

## Myeloid-derived suppressor cells

Myeloid-derived suppressor cells (MDSCs) comprise a heterogeneous group of immature myeloid cells characterized by their co-expression of CD11b and GR1 (184). In mice, two large populations can be distinguished, called polymorphonuclear (PMN)-MDSCs and monocytic (MO)-MDSCs (Figure 1). PMN-MDSC can be defined as CD11b^+^Ly6G^+^Ly6C^int^ cells with high production of ROS, while MO-MDSC on the other hand are defined as CD11b^+^Ly6G^−^Ly6C^high^ cells with high NO production (185, 186). In humans, MDSCs comprise three populations, a PMN-MDSC population identified by a CD14^−^CD11b^+^CD15^+^ (or CD66^+^) profile, a MO-MDSC population defined by a CD14^+^CD11b^+^HLA-DR^low/−^CD15^−^ phenotype and a population of “early stage MDSCs” or eMDSCs identified through the HLA-DR^−^/CD33^+^Lin^−^ profile (with Lin being CD3/14/15/19/56) (184). The presence of MDSCs is not restricted to cancer, but can occur in every form of chronic inflammation, including pathogenic infection (187), autoimmune diseases (188), and Alzheimer's disease (189). Their main role during inflammation is to temper the immune response in order to protect the body from tissue damage that can be caused by a prolonged and uncontrolled immune response (6, 190).

Tumor-associated MDSCs arise in the TME as the result of two groups of overlapping signals. On one hand, the presence of factors, such as GM-CSF, G-CSF, and M-CSF causes expansion of immature myeloid cells. On the other hand, a wide range of pro-inflammatory factors, e.g., PGE_2_, TNF, IL-1β, IL-6, S100A8, S100A9, IFNγ, IL-4, IL-10, and IL-13 secreted by cancer cells and leukocytes residing in the tumor inhibit the differentiation of myeloid progenitors and enhance their suppressive capacity (191). During cancer progression, MDSC levels do not only rise in the TME, but also increase in the spleen (192) and bone marrow (193), where they exert inhibitory functions on the immune system. However, the MDSCs in the TME were shown to exhibit higher immunosuppressive capacities than the peripheral MDSCs from the spleen (194) or bone marrow (193). In the TME of most cancer types, the PMN-MDSC fraction makes up around 80% of the total MDSC (6), with most of the MO-MDSC rapidly differentiating into TAMs (47).

In the TME, MDSCs exhibit different tumor-promoting and immunosuppressive functions and hence correlate with poor prognosis in cancer patients (195). The tumor-promoting functions comprise (i) remodeling of the TME (196), (ii) induction of (lymph)angiogenesis (196), (iii) promotion of metastasis (197), (iv) inhibition of cellular senescence (198), (v) suppression of T-cell function and migration (199, 200) and (vi) resistance to chemo-and immunotherapy (201–203). It is important to note that the immunosuppressive activity of MDSCs is not limited to a single mechanism, with MDSCs engaging several mechanisms throughout the progression of the tumor (6, 204–206), including; (i) expansion of Tregs (207), (ii) expression of galectin-9 on the MDSC surface, resulting in T-cell apoptosis (208), (iii) inhibition of NK cells through membrane-bound TGFβ1 (209), (iv) the secretion of ROS [${\text{O}}_{2}^{-}$, H_2_O_2_ and peroxynitrite (OONO^−^)](210, 211), (v) expression of enzymes involved in amino acid catabolism, like Arginase-I and IDO, collectively inhibiting T-cell proliferation (212, 213), and (vi) secretion of S100A8 and S100A9, resulting in the recruitment of more MDSCs and inhibition of dendritic cell maturation (214, 215).

Treatments targeting MDSCs in the TME aim to (i) reduce the number of MDSCs via their elimination or inhibition of recruitment or (ii) induce “re-education” or differentiation of these cells into anti-tumoral cells.

### Elimination of MDSCs or inhibition of MDSC recruitment

In order to counteract the immunosuppressive actions of MDSCs, many depletion strategies have been applied (Table 1). The use of the chemotherapeutic agents gemcitabine, 5-fluorouracil and cisplatin, is able to eliminate MDSCs in murine tumors by inducing their apoptosis (216–218). As mentioned above, S100A9 is one of the central inflammatory mediators promoting MDSC recruitment. Accordingly, peptibodies against S100A9 led to reduced MDSC recruitment in tumor-bearing mice (219). Tyrosine kinase inhibitors, such as ibrutinib and sunitinib, respectively in mice and in humans, have also been shown to decrease tumor growth and decrease the numbers of MDSCs present in the TME (221, 225). Interestingly, the antidiabetic drug phenformin has been recently shown to selectively deplete PMN-MDSCs in the TME in mouse models of melanoma through the activation of AMPK (226). Activation of TRAIL receptor 2 (TRAIL-R2, also known as DR5) using an agonist antibody provides a more selective approach to induce MDSC apoptosis due to high expression of TRAIL-R2 on MDSCs (231). The TRAIL-R2-targeting antibody has already progressed to a phase I clinical trial, which demonstrated efficient depletion of MDSCs (particularly PMN-MDSCs) in the blood of patients with various solid tumor types (224). Interestingly, however, only a subset of patients showed a decrease of MDSCs in the tumor microenvironment (224).

**Table 1:** Myeloid-derived suppressor cell depletion or recruitment inhibition strategies in murine cancer models and patients.

**Tumor model**	**Treatment**	**Target**	**Amount/type of MDSC**	**Outcome**	**Reference**
Mouse Lymphoma/melanoma	Gemcitabine-loaded nanopatricles	DNA synthesis	MO-MDSCs depletion	Attenuated immune suppression	(216)
Mouse Melanoma	5-Fluorouracil	DNA-synthesis	MDSCs depletion	Induced CD8^+^ T-cell response	(217)
Mouse Melanoma	Cisplatin	DNA-synthesis	MDSCs depletion	Partially abrogated immune suppression	(218)
Mouse Thymoma	Pep-H6 Pep-G3	S100A9	MDSC depletion	Retardation tumor growth	(219)
Mouse Breast carcinoma	SAR131675	VEGFR	Prevents MDSC accumulation + M1-like TAM differentiation	Reduced tumor growth and metastasis	(220)
Mouse Melanoma	Ibrutinib	Bruton's tyrosine kinase	MDSC reduced	Enhanced the efficacy of anti-PD-L1	(221)
Mouse HPV-expressing TC-1 cells	Anti-IL6R mAb	IL6	MDSC reduced	Reduced tumor growth	(222)
Mouse HPV-expressing TC-1 cells	S31	STAT3	MDSC reduced	Reduced tumor growth	(222)
Mouse Lung carcinoma	Anti-PI3Ky Integrin α_4_ mAb or KO mice for both	PI3Ky Integrin α_4_	Prevents MDSC accumulation	Reduced tumor growth	(223)
Mouse Melanoma	Phenformin (+anti-PD1)	Mitochondrial complex 1 of the respiratory chain (+ PD1)	PMN-MDSC depletion in spleen	Reduced tumor growth	(226)
Mouse Sarcoma	GW2850 PLX3397	CSF1R	Prevents MO-MDSC accumulation	Reduced tumor growth	(227)
Mouse Colorectal carcinoma	CD11b KO	CD11b	Decreased MDSC accumulation	Reduced tumor growth	(228)
Mouse Breast carcinoma Colon carcinoma	Entinostat (+ anti-PDL1 + anti-CTLA4)	Class I HDAC	MDSC inhibition	Reduced tumor growth	(229)
Human Head and neck squamous carcinoma	Tadalafil	PDE-5	Decreased MDSC circulating	Reversed immune suppression	(230)
Human Multiple cancers	DS-8273a (TRAILR2 agonist)	TRAILR2	MDSC depletion	NA	(224)
Human Renal cell carcinoma	Sunitinib	Multitargeted tyrosine kinase inhibitor	MDSC reduced	Improved tumor-infiltrating lymphocytes	(225)

Since both MO-MDCSs and TAMs derive from monocytic precursors, many inhibitors described to reduce the abundance of TAMs (cfr partim Macrophages) can be used to inhibit MO-MDSC recruitment as well (Table 1). For instance, in mice the use of the CSF-1R inhibitors GW2850 and PLX3397, led to a reduced recruitment of MO-MDSCs in the TME (227). Aside from CSF-1R inhibitors, the inhibition of PI_3_Kγ or integrin α_4_ prevented the accumulation of MDSCs as well as the expression of immunosuppressive molecules in the TME of LLC tumors (223). Analogously, genetic deletion of integrin-αM (also known as CD11b) in mice resulted in decreased recruitment of PMN-MDSCs to colorectal carcinomas and led to reduced tumor burden and improved survival, establishing integrin-αM as an additional therapeutic target (228). Similar findings were observed after inhibition of the IL-6/STAT3 pathways, leading to a significant inhibition of MDSC expansion and tumor growth of the murine TC1 tumor model (222). Also in mice, SAR131675, an inhibitor of VEGFR-3, led to a reduction in the frequency of MDSCs in the tumor and in the spleen (220). In patients, the inhibition of phosphodiesterase 5 using tadalafil reduced peripheral MDSC numbers which was associated with an enhanced proliferative capacity of patient-derived T cells in head and neck squamous cell carcinoma (230). Epigenetic modulators are generally thought to primarily affect cancer cells through inducing reexpression of silenced genes often involved in antigen presentation, potentially leading to enhanced antitumor immunity. However, administration of 5-azacytidine and entinostat to inhibit DNA methyltransferases and class I HDAC enzymes, respectively, has been shown reduce circulating and tumor-infiltrating PMN-MDSC levels which led to improved responses to immune checkpoint blockade therapy in mice (229). Interestingly, entinostat but not 5-azacytidine markedly reduced the viability of MDSCs (229). Nevertheless, the exact mechanism by which epigenetic regulators exert their inhibitory function on MDSCs remains to be elucidated.

The interplay of MDSCs with mast cells has also been considered an interesting future target. While mast cells have been associated with allergic reactions, they have also been reported to play either an immunostimulatory or an immunosuppressive role in the TME, depending on the tumor type (232). In the tumor-promoting context, mast cells do not only secrete immunosuppressive cytokines, but are also involved in the recruitment of MDSCs (233). Therefore, targeting the recruitment/function of tumor infiltrating mast cells could lead to diminished recruitment of MDSCs to the TME. Only few depletion strategies have been employed, which are reviewed in Varricchi et al. (232). Hence, further research on mast cells as a potential target in cancer immunotherapy is still needed.

Although the inhibition of MDSC recruitment to the TME provides a promising strategy, it can also be of interest to promote the differentiation of MDSCs toward either mature myeloid cells with antigen-presenting and/or cytotoxic activity.

### Differentiation of MDSCs into anti-tumoral myeloid cells

A method to convert immunosuppressive MDSC to anti-tumoral myeloid cells might rely on TLR activation. For instance, the administration of a TLR7/8 agonist, resiquimod, led to the differentiation of bone marrow-derived MO-MDSC into F4/80^+^ macrophages and CD11c^+^ dendritic cells *in vitro* (234, 235). A recent study by Shayan et al. also demonstrated that the use of a TLR8 agonist in combination with the EGFR inhibitor cetuximab led to repolarization of monocytes toward an M1-like TAM phenotype and resulted in less MDSC-mediated suppression of T-cell activity *in vitro*. Furthermore, administration of the combination treatment was associated with a more immune-permissive TME in patients with head and neck squamous cell carcinoma (236). This however raises the question whether the differentiated monocytes were in fact MO-MDSCs that differentiated toward an anti-tumoral M1 TAM, as proposed in Wang et al. [2015] or whether the differentiation of monocytes toward M1-like TAMs overruled the suppressive actions of the MDSCs present in the TME (237).

Conversely, TLRs can also be involved in sustaining MDSC-mediated immune suppression. For instance, in pancreatic cancer in mice, TLR9 activation has been shown to induce MDSC proliferation *in vivo* and activate pancreatic stellate cells to display protumorigenic effects *in vitro* (238). Accordingly, activating TLR2 signaling in the murine EG7 lymphoma model via the Pam2CSK4 lipopeptide, leads to an increased immunosuppressive activity of MO-MDSCs as they further differentiate into protumoral macrophages (239). However, the administration of N6-(1-Iminoethyl)-L-lysine (L-NIL), an iNOS inhibitor, decreased the immunosuppressive effect, showing the therapeutic potential of Pam2CSK4 when used in combination with other therapeutic agents (239). Another ligand for TLR2, Hsp72, has also proven to activate and increase the suppressive capacities of MDSCs in murine lymphoma, mammary carcinoma and colon carcinoma models, and showed relevance in humans as the human tumor cell line TDE triggered the suppressive function of MDSCs in a Hsp72 dependent manner (240). Also Hsp90, a regulator of TLR4 signaling, showed to be involved in the induction of the suppressive capacities of MDSCs *in vitro* (241). Therefore, the use of TLRs in MDSC-based immunotherapy remains to be further investigated.

Interestingly, oral administration of yeast-derived whole β-glucan particles (WGP) activated the dectin-1 receptor, leading to reduced amounts of PMN-MDSC in the spleens and tumors of LLC and E0771 tumor-bearing mice and decreased their immunosuppressive properties *in vitro*. In an *in vitro* assay, the presence of WGP induced the differentiation of MO-MDSC into F4/80^+^ CD11c^+^ myeloid cells, serving as potent APCs and when injected intratumorally, WGP-treated MO-MDSCs were capable of inhibiting tumor growth in subcutaneously inoculated LLC (242).

Using the antibody 2aG4 against another therapeutic target, phosphatidylserine, also showed repolarization from M2-like TAMs to the M1-like phenotype, together with differentiation of MO-MDSCs into M1-like TAMs and dendritic cells *in vitro* (243). Interestingly, curcumin-based chemotherapy (docetaxel) showed to selectively eliminate the PMN-MDSCs, while sparing the MO-MDSC which then repolarized toward M1-like TAMs in a murine 4T1 mammary carcinoma model (244).

A study performed on *in vitro* generated MDSCs co-cultured with the human A375 melanoma cell line demonstrated a shift of the MDSC phenotype toward a profile associated with immunostimulatory dendritic cells, through the inhibition of macrophage migration inhibitory factor (MIF) with 4-iodo-6-phenylpyrimidine (245). However, these results remain to be confirmed *in vivo* before MIF inhibition can be further explored in a therapeutic setting. Shen et al. also witnessed a similar shift of the immunosuppressive MDSCs toward a more immunostimulatory myeloid cell type in response to tasquinimod, a quinoline−3-carboxyamide analog with anti-angiogenic properties when administered to mice injected with either castration-resistant prostate cancer or melanoma cells (246).

Moreover, the administration of axitinib, a small molecule tyrosine kinase inhibitor of VEGFR-1/2/3, reduced the immunosuppressive activity of splenic and tumor-infiltrating MO-MDSCs besides its anti-angiogenic effect. Moreover, MO-MDSCs from axitinib-treated tumors in mice were able to stimulate T-cell activation, suggesting a phenotype switch from immunosuppressive to antigen-presenting activity (247).

## Concluding remarks

The use of tumor-associated immune cells unlocks an interesting field of potential therapies in the fight against cancer. Severe side effects inflicted by conventional therapies are overcome as the body's own immune system engages in specific anti-tumoral immune responses. Moreover, the genomic stability of tumor-associated immune cells as opposed to the high genetic plasticity and heterogeneity of cancer cells, decreases the risk of developing resistance against immunotherapies.

Still many hurdles are to be overcome in order to completely rely on the immune system to ensure specific and long-term immune responses against tumors. The observation that the abundance of myeloid cell (sub)population can differ substantially between tumor types (248, 249), urges for the verification of their therapeutic potential in distinct tumor models. Additionally, high variability in the frequency of distinct myeloid cell subsets is also witnessed between patients with the same tumor type (30–32). As highlighted in this review, clinical translation of some of the therapeutic strategies targeting myeloid cells is ongoing. The observations above have two crucial implications for future translational efforts. Firstly, murine models will likely fail to predict therapeutic responses to myeloid cell-based therapies in patients with cancer, as tumor models in mice, particularly transplantable ones, show rapid progression and low variability in their immune microenvironment. Thus, there is an urgent need for the development and application of more advanced pre-clinical models that recapitulate the patient-to-patient heterogeneity of the tumor immune microenvironment. Secondly, similar to ICIs, likely not all patients will benefit from myeloid cell-targeted therapies. Thus, it will be essential to investigate the differences between the responder and non-responder populations in order to identify biomarkers predicting therapy response. Due to the highly patient-specific nature of tumor antigens and the tumor immune microenvironment, the future myeloid-cell targeted therapies will have to be integrated in combination therapies tailored to each patient, in which adoptive T-cell transfer, ICIs, co-stimulatory molecules, low-dose chemo-or radiotherapy are combined with the (re)activation of tumor-associated myeloid cells.

## Author contributions

EC wrote the manuscript draft. AM designed the figures HV revised the manuscript draft. MK and DL supervised and wrote the final manuscript with input from all authors.

### Conflict of interest statement

The authors declare that the research was conducted in the absence of any commercial or financial relationships that could be construed as a potential conflict of interest.
